# Brain white matter development in 8-year-old children is associated with maternal mental health during pregnancy

**DOI:** 10.3389/fnhum.2025.1603022

**Published:** 2025-06-18

**Authors:** Yali Huang, Timothy R. Koscik, Aline Andres, Jayne Bellando, Charles M. Glasier, Adhitya Ram, Xiawei Ou

**Affiliations:** ^1^Department of Radiology, University of Arkansas for Medical Sciences, Little Rock, AR, United States; ^2^Department of Pediatrics, University of Arkansas for Medical Sciences, Little Rock, AR, United States; ^3^Arkansas Children’s Research Institute, Little Rock, AR, United States; ^4^Arkansas Children’s Nutrition Center, Little Rock, AR, United States; ^5^University of Arkansas, Fayetteville, AR, United States

**Keywords:** prenatal maternal mental health, children’s neurodevelopment, limbic system white matter, diffusion tensor imaging (DTI), diffusion kurtosis imaging (DKI), neurite orientation dispersion and density imaging (NODDI)

## Abstract

**Background:**

Maternal mental health during pregnancy can influence fetal brain development, yet its long-term effects remain unclear. This study investigates the association between prenatal maternal depression and anxiety symptoms and white matter microstructure in the limbic system of 8-year-old children.

**Methods:**

Fifty-one healthy pregnant women and typically developing 8-year-old children dyads were included in this prospective and longitudinal study. Maternal depression and anxiety symptoms were assessed at 12, 24, and 36 weeks of gestation using the Beck Depression Inventory-II (BDI-II) and State–Trait Anxiety Inventory (STAI). Their children underwent a brain MRI examination at age 8 years with multi-shell diffusion imaging analyzed using diffusion tensor imaging (DTI), diffusional kurtosis imaging (DKI), and neurite orientation dispersion and density imaging (NODDI) models for a multi-aspect evaluation of microstructural development. Key diffusion metrics (FA: fractional anisotropy; MD: mean diffusivity; AD: axial diffusivity; RD: radial diffusivity; MK: mean kurtosis; AK: axial kurtosis; RK: radial kurtosis; NDI: neurite density index; ODI: orientation dispersion index; FWF: free water fraction) were extracted from the limbic system white matter structures including cingulum, fornix, and uncinate fasciculus, which are closely associated with emotional and motivational processes.

**Results:**

Higher maternal depression symptom scores were associated with lower FA (*R* = –0.3126, *p* = 0.0305, in CGH.R; *R* = –0.3025, *p* = 0.0366, in FXC.R) and MK (*R* = –0.3284, *p* = 0.0227, in CGG.R) and higher MD (*R* = 0.2879, *p* = 0.0472, in CGH.R) and RD (*R* = 0.3451, *p* = 0.0163, in CGH.R; *R* = 0.3456, *p* = 0.0161, in FXC.R) in predominately right-hemisphere limbic tracts. Higher maternal anxiety symptom scores were associated with increased MD (*R* = 0.2897, *p* = 0.0458, in FXC.L; *R* = 0.2859, *p* = 0.0488, in UF.L) and RD (*R* = 0.3168, *p* = 0.0283, in FXC.L), decreased NDI (*R* = –0.3787, *p* = 0.0079, in FXC.L; *R* = –0.3422, *p* = 0.0173, in UF.R), and increased AK (*R* = 0.3154, *p* = 0.029, in UF.L) in predominately left-hemisphere limbic tracts.

**Conclusion:**

Our findings suggest that maternal depression and anxiety during pregnancy may have long-lasting impacts on offspring white matter microstructure maturation in the limbic system. This highlights the need for prenatal mental health screening and potential interventions to promote brain development and support optimal neurodevelopmental outcomes in children.

## 1 Introduction

Depression and anxiety are highly prevalent among pregnant women. An analysis of data from the 2010–2019 National Health Interview Survey shows depression prevalence rates of 27.2 to 40.6% for different racial groups among pregnant women aged 18–44 (Sulley et al., 2023). In parallel, gestational anxiety disorders are estimated to occur in approximately 15–20% of pregnancies (Fawcett et al., 2019). Emerging research suggests that prenatal maternal mental health may have long-lasting effects on offspring neurodevelopment, particularly in white matter microstructures (Lebel et al., 2016; Wu et al., 2024). Our previous study demonstrated significant associations between maternal anxiety and depressive symptoms in the third trimester of pregnancy and reduced neonatal brain white matter integrity, as evidenced by decreased fractional anisotropy (FA) values, particularly affecting white matter regions in the frontal lobe, middle frontal gyrus, and limbic system (Graham et al., 2020). Other studies also showed that higher maternal anxiety and depression levels were associated with reduced white matter integrity in the right frontal lobe regions (Dean et al., 2018), lower FA and AD in the right amygdala (Rifkin-Graboi et al., 2013), as well as variations of FA across limbic and prefrontal regions (Rifkin-Graboi et al., 2015). Ross et al. found that by 2–3 years of age, children exposed to prenatal depression showed changes in white matter integrity, including increased FA and decreased mean diffusivity (MD) and radial diffusivity (RD), particularly in commissural and projection fiber regions (Roos et al., 2022). EI Marroun et al. reported that maternal depressive symptoms during pregnancy were associated with white matter microstructural alterations in children aged 6–9 years, specifically increased MD in the uncinate fasciculus and decreased FA and increased MD in the cingulum bundle (El Marroun et al., 2018). Wu et al. reviewed multiple MRI-based studies showing that when mothers experience elevated psychological stress during pregnancy, the fetus or newborn show volumetric or microstructural abnormalities in key brain regions such as the hippocampus, amygdala, and cingulate gyrus (Wu et al., 2024). These findings showed that prenatal maternal mental health may impact child neurodevelopment, particularly in white matter structures involved in motivation, emotion, learning, and memory within the limbic system (Wu et al., 2024; El Marroun et al., 2018; Gray, 1983).

Studies have emphasized white matter microstructural integrity as an important biomarker for mental health issues and proposed white matter trajectory models to evaluate neurodevelopment and neurodegeneration (Kochunov and Hong, 2014; Peters and Karlsgodt, 2015). Diffusion MRI is sensitive in evaluating white matter microstructural development, and different advanced diffusion model (DTI, DKI, and NODDI) can provide valuable information on microstructural integrity from a different perspective. DTI is widely used and sensitive to white matter, but assumes Gaussian diffusion, limiting its capacity to capture complex tissue architectures (Paydar et al., 2014). DKI extends DTI by incorporating non-Gaussian diffusion, thereby enhancing sensitivity to subtle microstructural heterogeneity (Paydar et al., 2014; Shi et al., 2019). NODDI, an advanced multi-compartment dMRI model, refines brain microstructure characterization by modeling neurite density, fiber orientation dispersion, and free water content (Huang et al., 2022; Zhang et al., 2012; Lynch et al., 2020). Combining these three techniques will provide a comprehensive, more nuanced, and biologically meaningful understanding of white matter microstructural development.

Mounting evidence shows that the limbic system—including key tracts such as the cingulum, fornix, and uncinate fasciculus—plays a pivotal role in emotional regulation, motivation, learning, and memory, rendering it particularly vulnerable to anxiety and depression (Gray, 1983; Torrico and Abdijadid, 2023; Van Velzen et al., 2020; Santos et al., 2018; Schmaal et al., 2016). Moreover, maternal mental health issues during pregnancy can significantly shape the development of these limbic pathways in offspring, potentially heightening their risk for future mental health challenges (Lebel et al., 2016; Wu et al., 2024; Graham et al., 2020; Dean et al., 2018; Rifkin-Graboi et al., 2013; Rifkin-Graboi et al., 2015; Roos et al., 2022; El Marroun et al., 2018). Given these considerations, understanding how prenatal maternal anxiety and depression may predispose children to alterations in limbic white matter microstructure is critical. Therefore, in this study, we focus on examining the relationships between maternal mental health during pregnancy and the white matter microstructural integrity of the vital limbic system in 8-year-old children. We hypothesized that higher maternal anxiety and depression symptoms during pregnancy will be associated with lower white matter microstructural development in these regions.

## 2 Materials and methods

### 2.1 Study design and participants

This is part of a prospective and longitudinal study (the *Glowing Follow Up* study, *NCT03108001*), which is a follow up of a previous prospective, observational study (*the Glowing study,* *clinicaltrials.gov* *NCT01131117*). All study procedures were approved by the institutional review board of the University of Arkansas for Medical Sciences, and appropriate consents/assents were obtained from study participants. In the *Glowing* study, pregnant women were enrolled and their newborns were initially followed up until age 2 years. Inclusion criteria for the pregnant women in the *Glowing* study were: pre-pregnancy body mass index (BMI) of 18.5–35, second parity, singleton pregnancy, ≥ 21 years old, conceived without assisted fertility treatments. Exclusion criteria for the pregnant women were preexisting medical conditions, medical complications during pregnancy, use of medications during pregnancy known to influence fetal growth, and tobacco and/or alcohol use. For their offspring, inclusion criteria were healthy and full-term at birth, while exclusion criteria were medications and medical conditions known to influence child growth and development. Participants who were enrolled in the original *Glowing* study and agreed to be contacted for future studies were contacted to participate in the *Glowing Follow Up* study. In this follow up study, children underwent an MRI examination of their brain and neurodevelopment assessments at age 8 years. A total of 67 mother/child dyads had both valid mental health assessments during pregnancy and a successful brain diffusion MRI scan at age ~8 years; 16 children were scanned after a scanner system upgrade which resulted in significant data harmonization issues for the diffusion MRI scans which have not been resolved and therefore excluded from this report; 51 mother/child dyads are included in this study report. Table 1 shows the demographic information of the study participants.

**Table 1:** Demographic information for the research subjects included in this study.

Group	Age(mean)	Age (std)	Age (range)	Sex
Children	8.13	0.14	8.00–8.74	28 M/23F
Mother	30.29	3.70	23.61–42.36	51F

### 2.2 Prenatal maternal depression and anxiety symptoms

**Figure 1:**
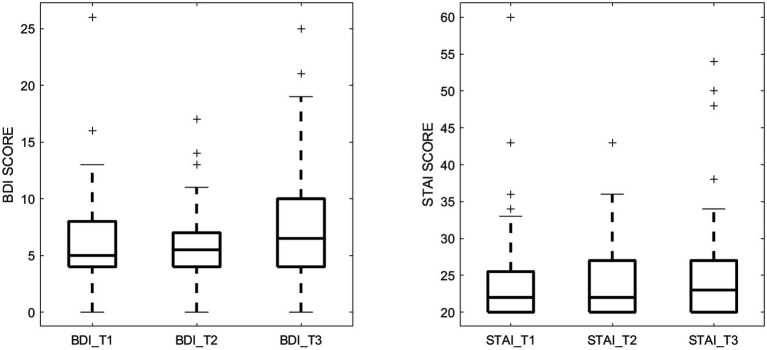
Maternal depression symptom scores (measured by BDI) and anxiety symptom scores (measured by STAI) during the 3 pregnancy trimesters (T1, T2, and T3).

Mental health of the pregnant women, specifically depression and anxiety symptoms during pregnancy, was assessed at three time points throughout the pregnancy─12 weeks (T1), 24 weeks (T2), and 36 weeks (T3) of gestation by trained psychological examiners. Depression symptoms were measured using the Beck Depression Inventory-II (BDI-II) (Beck et al., 1996), a validated and widely used 21-item self-report questionnaire, where each item is scored 0–3, resulting in a total score ranging from 0 to 63, with higher scores showing greater depression symptoms. State anxiety symptoms were assessed using the State–Trait Anxiety Inventory (STAI) (Speilberger et al., 1983), a 20-item self-report tool with each item scored 1–4, and total scores ranging from 20 to 80, where higher scores showing greater state anxiety symptoms. The measured BDI and STAI scores for the study cohort are presented in Figure 1.

### 2.3 Brain MRI data acquisition

At ~8 years of age, each child underwent an MRI scan of the brain without sedation using a PRISMA 3 T MRI scanner (Siemens Healthneers, USA) and a 20-channel head coil. The imaging protocol included a sagittal MPRAGE 3D T1-weighted sequence with TR 2,400 ms, TE 2.22 ms, 8 degree flip angle, turbo factor of 256, 192 sagittal slices, and a resolution of 0.8 mm x 0.8 mm x 0.8 mm, and an axial Multiband EPI pulse sequence for diffusion MRI data acquisition with TR 3,230 ms, TE 89.2 ms, 92 slices, resolution 1.5 mm x 1.5 mm x 1.5 mm, multiband factor 4. A multi-shell diffusion scheme was used with b-values of 0, 1,500, and 3,000 s/mm^2^, sampled using 7, 47, and 46 diffusion directions, respectively. Diffusion-weighted images at all b-values were acquired in both anterior–posterior (AP) and posterior–anterior (PA) phase-encoding directions to enable susceptibility distortion correction using the TOPUP approach.

Although some advanced diffusion protocols (such as early DKI protocols) often limited maximum b-values to 2000 s/mm^2^, recent studies support the inclusion of higher b-values—up to 3,000 s/mm^2^—for improved sensitivity to microstructural complexity in brain tissue without violating model assumptions (Chou et al., 2017; Olson et al., 2018; Bano et al., 2024). This b-value selection reflects a balance between increased diffusion contrast and reduced signal-to-noise ratio (SNR), which can be mitigated by modern acquisition techniques (Yan et al., 2013; Chuhutin et al., 2017; Kuo et al., 2018; Ezequiel Farrher and Shah, 2012).

### 2.4 Imaging data preprocessing

The diffusion imaging data were preprocessed using MRtrix3, FSL, and ANTs (Tournier et al., 2019; Jenkinson et al., 2012; Avants et al., 2009). All diffusion-weighted datasets underwent a standardized preprocessing workflow as follows. First, the raw diffusion images were denoised using a Marchenko-Pastur principal components analysis (MP-PCA) approach to minimize thermal noise. Next, Gibbs ringing artifacts were removed prior to correcting for geometric and motion-related distortions. Specifically, head motion, eddy current, and susceptibility-induced distortions were addressed using FSL’s Topup (for dual AP-PA acquisitions) followed by Eddy to correct for motion and remaining eddy currents. Subsequently, bias field correction was applied to reduce low-frequency intensity inhomogeneities across the image volume. Finally, global intensity normalization was performed by normalizing the input diffusion-weighted MRI images to have the same B0 white matter median value to remove intensity variations due to T2-weighting and RF inhomogeneity. This preprocessing pipeline ensures consistent image quality and facilitates robust downstream diffusion modeling and parameter mapping. To extract diffusion parameters, we utilized three diffusion models of white matter microstructure. DTI metrics were computed using the MRtrix3, yielding FA, axial diffusivity (AD), RD, and MD (Tournier et al., 2019). DKI model fitting was conducted with DIPY toolbox,^1^ providing mean kurtosis (MK), axial kurtosis (AK), and radial kurtosis (RK) (Henriques et al., 2021). NODDI parameters were derived using the open-source tool AMICO,^2^ generating neurite density index (NDI), orientation dispersion index (ODI), and free water fraction (FWF) (Daducci et al., 2015). Together, these models enabled a detailed and multi-faceted assessment of brain diffusion properties, offering a refined characterization of white matter microstructure.

### 2.5 Region of interest (ROI) extraction

**Figure 2:**
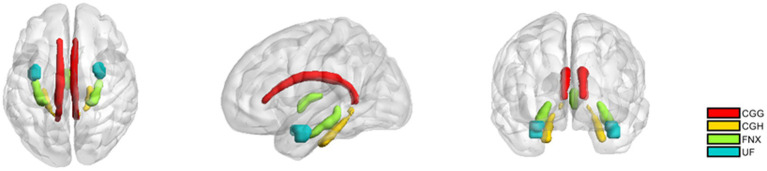
The white matter regions of interest in the limbic system. CGG: Cingulum (cingulate gyrus); CGH: Cingulum (hippocampus); fornix (FNX); uncinate fasciculus (UF). The definitions of these regions come from the JHU atlas.

The limbic system white matter regions of interest (ROIs) were extracted using the Johns Hopkins University (JHU) white matter atlas (Mori et al., 2008). Specifically, the white matter ROIs encompassed the cingulum gyrus (CGG) and the cingulum hippocampus (CGH), fornix (FNX), and uncinate fasciculus (UF) were reconstructed and average diffusion parameters within each ROI were calculated for each subject. A visual representation of the limbic system white matter regions studied is provided in Figure 2.

### 2.6 Statistical analysis

To examine the relationships between prenatal maternal mental health and child white matter microstructure at 8 years of age, we conducted Spearman correlation analyses between maternal BDI and STAI scores (at T1, T2, and T3) and the diffusion metrics (FA, MD, AD, RD, MK, AK, RK, NDI, ODI, and FWF) extracted from the limbic system white matter ROIs. Child age and sex were included as covariates to control for potential confounding effects in the analysis. Spearman rank correlation coefficients (*ρ*) were used to assess associations between maternal symptom scores and diffusion MRI metrics. This non-parametric method was chosen due to the non-normal distribution of several imaging variables and to account for potential non-linear but monotonic relationships between psychological measures and neuroimaging indices.

## 3 Results

### 3.1 Relationships between BDI-II and diffusion metrics

#### 3.1.1 Time points

At each of the three trimesters during pregnancy (T1, T2, T3), significant correlations between maternal depression symptoms and child white matter microstructure were observed (4 for T1; 5 for T2 and 7 for T3), as shown in Table 2.

**Table 2:** Significant correlation between maternal depression symptom scores (measured by BDI) during the 3 pregnancy trimesters (T1, T2, and T3) and children brain diffusion imaging parameters at age 8 years.

Time point	Diffusion model	Diffusion metric	Brain region	*R*	*P*
T1	DTI	RD	UF.R	0.2945	0.0469
T1	DKI	MK	CGH.R	0.2972	0.0449
T1	NODDI	NDI	FNX.R	−0.2953	0.0463
T1	NODDI	NDI	UF.R	−0.3453	0.0188
T2	DKI	MK	CGG.R	−0.3268	0.0234
T2	NODDI	NDI	CGG.R	−0.2859	0.0489
T2	NODDI	FWF	CGG.L	−0.2977	0.0399
T2	NODDI	FWF	CGG.R	−0.3101	0.032
T2	NODDI	FWF	UF.L	−0.3	0.0383
T3	DTI	FA	CGH.R	−0.3126	0.0305
T3	DTI	FA	FNX.R	−0.3025	0.0366
T3	DTI	MD	CGH.R	0.2879	0.0472
T3	DTI	RD	CGH.R	0.3451	0.0163
T3	DTI	RD	FNX.R	0.3456	0.0161
T3	DKI	MK	CGG.R	−0.3284	0.0227
T3	NODDI	FWF	CGG.R	−0.294	0.0425

#### 3.1.2 Diffusion modality

**Figure 3:**
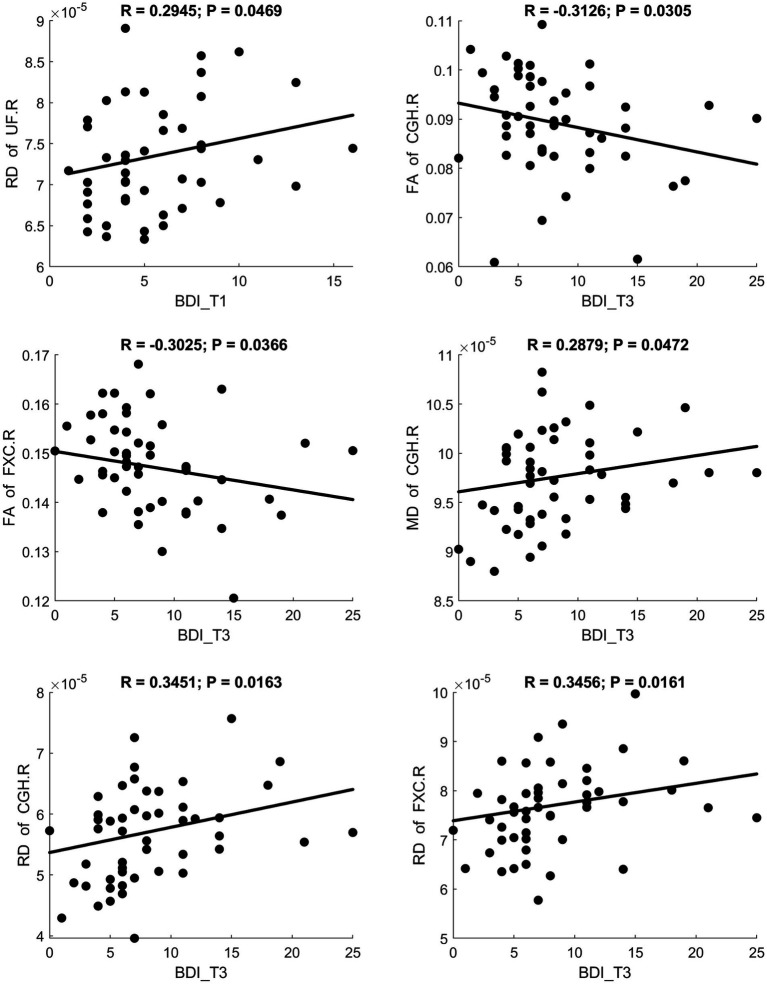
Significant correlation between maternal depression symptom scores (measured by BDI) during the 3 pregnancy trimesters (T1, T2, and T3) and children brain diffusion imaging parameters (measured by DTI) at age 8 years.

**Figure 4:**
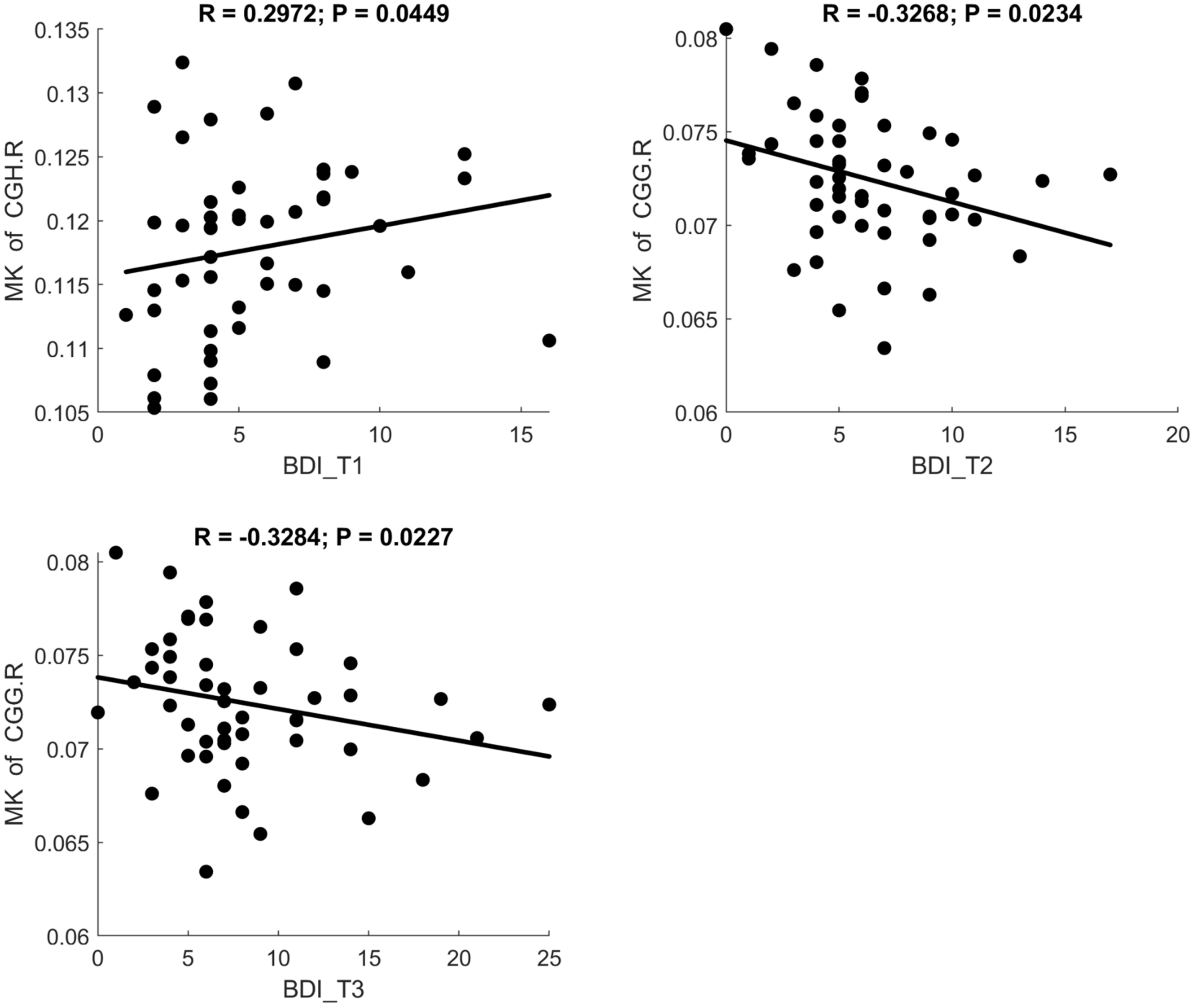
Significant correlation between maternal depression symptom scores (measured by BDI) during the 3 pregnancy trimesters (T1, T2, and T3) and children brain diffusion imaging parameters (measured by DKI) at age 8 years.

**Figure 5:**
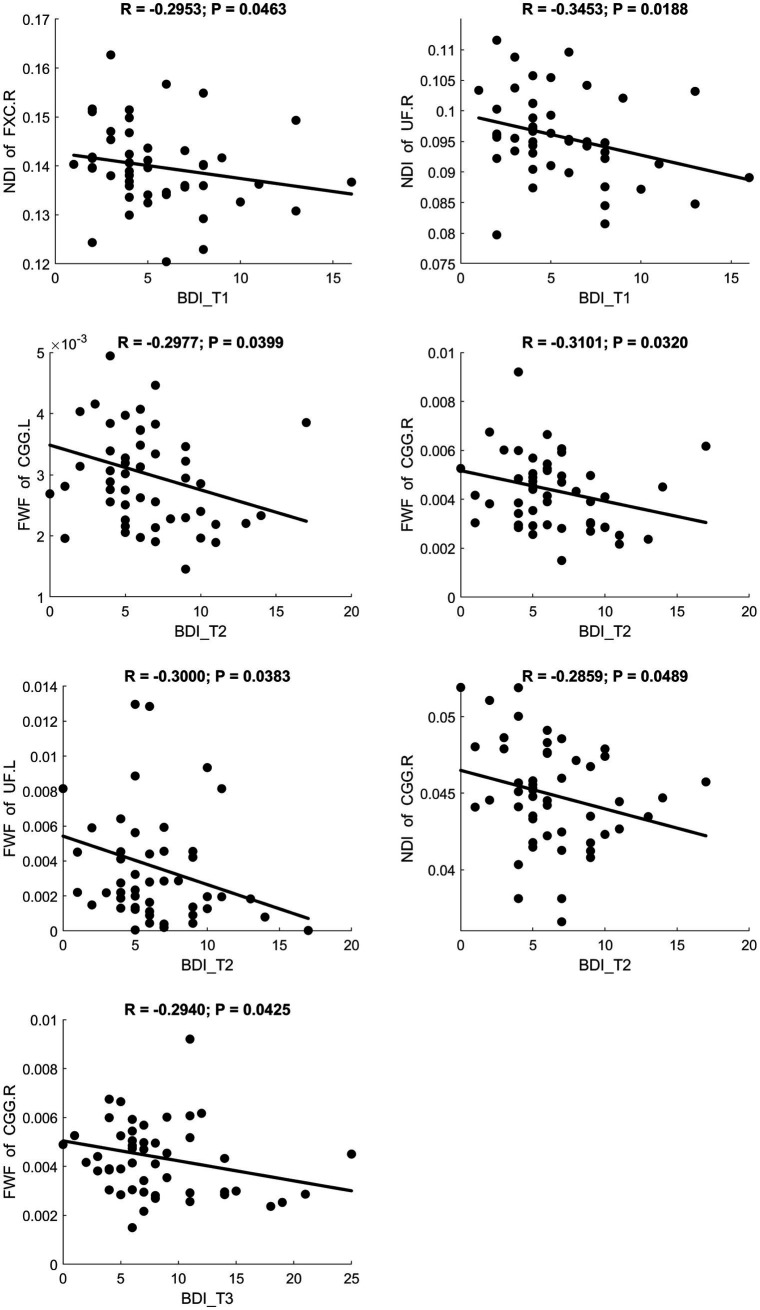
Significant correlation between maternal depression symptom scores (measured by BDI) during the 3 pregnancy trimesters (T1, T2, and T3) and children brain diffusion imaging parameters (measured by NODDI) at age 8 years.

Fractional anisotropy showed a significant negative correlation with BDI scores, indicating reduced white matter fiber integrity in individuals with maternal depression. MD and RD showed significant positive correlations with BDI scores, suggesting increased diffusivity in white matter. A scatter plot depicting these relationships is presented in Figure 3. Additionally, MK and FWF showed significant negative correlations with BDI scores. Scatter plots illustrating these relationships are presented in Figures 4, 5.

#### 3.1.3 Brain regions

Significant results were predominantly observed in the right hemisphere white matter fiber tracts, specifically, 14 out of 16 significant relationships involved the limbic system in the right hemisphere, as shown in Table 2.

### 3.2 Relationships between STAI and diffusion metrics

#### 3.2.1 Time points

At the three trimesters during pregnancy (T1, T2, T3), significant correlations between maternal anxiety symptoms and child white matter microstructures were also observed (3 for T1, 3 for T2 and 6 for T3), as shown in Table 3.

**Table 3:** Significant correlation between maternal anxiety symptom scores (measured by STAI) during the 3 pregnancy trimesters (T1, T2, and T3) and children brain diffusion imaging parameters at age 8 years.

Time point	Diffusion model	Diffusion metric	Brain Region	*R*	*P*
T1	DTI	MD	CGH.L	−0.4379	0.0023
T1	DKI	MK	CGH.R	0.3308	0.0247
T1	DKI	AK	CGH.L	0.427	0.0031
T2	DTI	AD	FNX.L	0.2953	0.0416
T2	NODDI	NDI	FNX.L	−0.4183	0.0031
T2	NODDI	FWF	UF.L	−0.3389	0.0185
T3	DTI	MD	FNX.L	0.2897	0.0458
T3	DTI	MD	UF.L	0.2859	0.0488
T3	DTI	RD	FNX.L	0.3168	0.0283
T3	DKI	AK	UF.L	0.3154	0.029
T3	NODDI	NDI	FNX.L	−0.3787	0.0079
T3	NODDI	NDI	UF.R	−0.3422	0.0173

#### 3.2.2 Diffusion modality

**Figure 6:**
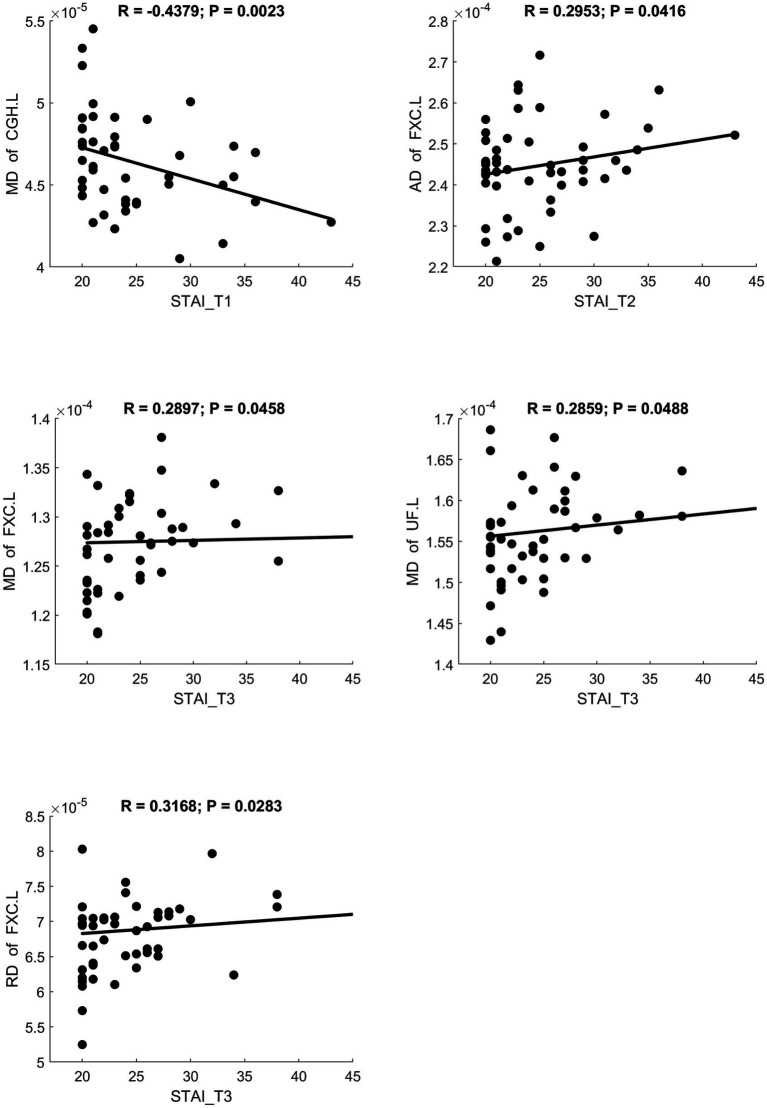
Significant correlation between maternal anxiety symptom scores (measured by STAI) during the 3 pregnancy trimesters (T1, T2, and T3) and children brain diffusion imaging parameters (measured by DTI) at age 8 years.

**Figure 7:**
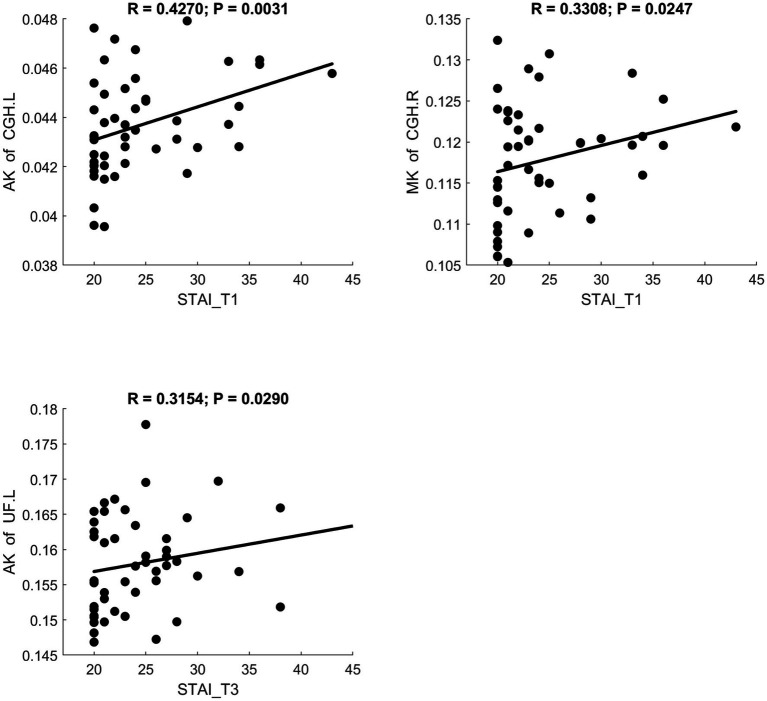
Significant correlation between maternal anxiety symptom scores (measured by STAI) during the 3 pregnancy trimesters (T1, T2, and T3) and children brain diffusion imaging parameters (measured by DKI) at age 8 years.

**Figure 8:**
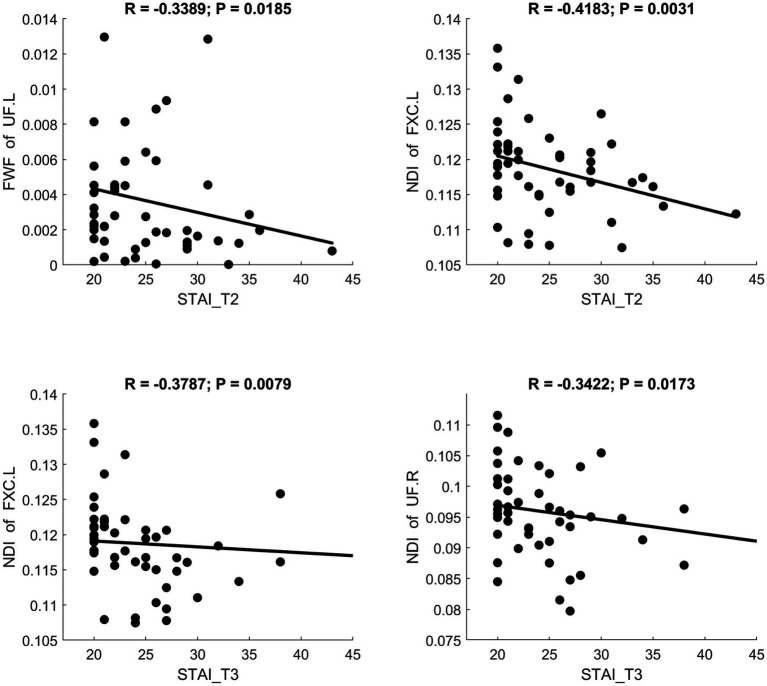
Significant correlation between maternal anxiety symptom scores (measured by STAI) during the 3 pregnancy trimesters (T1, T2, and T3) and children brain diffusion imaging parameters (measured by NODDI) at age 8 years.

MD and RD were significantly positively correlated with STAI scores. AK showed a significant positive correlation with STAI. NDI showed a significant negative correlation with STAI. Scatter plots illustrating these relationships are presented in Figures 6–8, respectively.

#### 3.2.3 Brain regions

Significant results were predominately observed in the left hemisphere white matter fiber tracts, specifically, 10 out of 12 significant relationships involved the limbic system in the left hemisphere, as shown in Table 3.

## 4 Discussion

This study aimed to investigate relationships between maternal mental health during pregnancy and long-term brain development of offspring. Specifically, we tracked the white matter microstructural development of 51 eight-year-old children and identified significant correlations between child brain white matter microstructural properties and their mothers’ depression (BDI) and anxiety (STAI) symptoms assessed at different gestational stage (12 weeks, 24 weeks, and 36 weeks of pregnancy, respectively). By utilizing advanced diffusion imaging and applying DTI, DKI, and NODDI models, we extracted FA, MD, AD, RD, MK, AK, RK, NDI, ODI, and FWF parameters, with a particular focus on limbic white matter regions—namely, the cingulum (both cingulate gyrus and hippocampus), fornix, and uncinate fasciculus. Overall, our results showed that maternal depression symptoms correlated predominantly with white matter microstructural integrity in the right brain, whereas maternal anxiety symptoms correlated predominantly with white matter microstructural integrity in the left brain. Our findings suggest that prenatal maternal mental health may be associated with changes in the development of fetal limbic system white matter, which may persist to later ages in childhood.

There are some similarities between our BDI/STAI and brain correlation analyses findings. Both BDI and STAI showed positive correlations with MD and RD measured by DTI. These results suggest that higher maternal psychological stress in pregnancy may reduce tissue density or levels of myelination, thereby increasing water diffusivity and compromising the organization of white matter. Both BDI and STAI also showed negative correlations with NDI measured by NODDI, indicating that maternal psychological stress may inhibit optimal neuronal growth or myelination in the developing brain and therefore impact neurite density.

There are also some interesting differences between our BDI/STAI and brain correlation analyses findings. In particular, maternal depression symptoms measured by BDI were more prominently related with right-sided limbic white matter (e.g., right cingulum and right fornix), which are crucial for emotional regulation and memory processing, as shown in Table 2. In contrast, maternal anxiety symptoms measured by STAI was more prominently related with left-sided limbic white matter, as shown in Table 3, which are crucial for language, cognitive processing of emotions, and possibly internal worry loops. Our finding that maternal depression significantly correlated with right hemisphere limbic white matter in children is consistent with literature findings reporting abnormal right hemisphere brain function involved in depression. According to Hecht, depression is often characterized by hyperactivation of the right hemisphere and relative hypoactivation of the left hemisphere, manifesting as amplified negative emotional processing, pessimistic thinking, and enhanced self-focus or rumination (Hecht, 2010). Belden et al. found that children with a smaller insula in the right hemisphere of the brain — related either to depression or excessive guilt — were more likely to have recurrent episodes of clinical depression as they got older (Belden et al., 2015). Likewise, Li et al. reported increased regional homogeneity in multiple right-hemisphere regions—such as frontal, temporal, and cingulate regions—in depressed patients compared to healthy controls, indicating a predominance of right-hemisphere involvement in depression (Li et al., 2018). Our findings of maternal anxiety correlation with left-sided limbic white matter in children echoes Blackmon et al. who found that higher subclinical anxiety levels in healthy adults were associated with smaller left amygdala volumes but increased cortical thickness in the left lateral orbitofrontal cortex and temporoparietal junction (Blackmon et al., 2011).

In addition to these lateralization patterns, our study combined DTI, DKI, and NODDI to capture multiple dimensions of white matter microstructural differences associated with maternal depression and anxiety. Numerous studies have reported the sensitivity of DTI parameters to white matter microstructural changes. Previous work by Paydar et al. has also shown that DKI metrics (e.g., MK, AK, RK) may detect subtle developmental changes from childhood into adolescence even beyond what FA reveals using DTI (Paydar et al., 2014). Meanwhile, Zhao et al. observed that NDI is especially sensitive to early changes in myelination and neurite density from birth through adolescence (Zhao et al., 2021). The combination of DTI/DKI/NODDI in the same study provided a comprehensive characterization of white matter microstructural changes in the limbic system associated with maternal depression and anxiety.

Despite the strength of longitudinal and prospective design and comprehensive characterization of maternal depression and anxiety throughout pregnancy and white matter microstructural development at age 8 years, our study has several limitations. First, the sample size is relatively small, limiting statistical power for more strict multiple comparison correction and the generalizability of results; larger cohorts should be considered in future research. Second, we only measured brain imaging data at one time point (age 8 years) after the newborn period, restricting our ability to capture potential developmental trajectories across childhood. Research MRI in younger children without sedation can be completed with high success rate and future study should implement longitudinal scans at multiple ages. Third, there are many other prenatal and postnatal factors which may also impact child white matter microstructural development and they are not included in this study as potential confounders. We cannot rule out the influence of genetic factors or postnatal environmental exposures. Maternal depression and anxiety may reflect broader psychosocial contexts, including parenting quality or ongoing maternal mental health, which were not assessed in this study. Our findings suggest correlation rather than causation, and interpretations should take into account the potential influences of both prenatal and postnatal factors. Future large-scale studies with comprehensive assessments of maternal and child mental and physical health, family environment, and lifestyle factors are warranted. Finally, although we focused on limbic white matter regions due to their known roles in emotion and memory that are impacted by mental health, many other brain networks and/or regions may potentially also show long-lasting changes associated with maternal mental health, and whole-brain analysis will be beneficial.

## 5 Conclusion

In summary, this study demonstrates that maternal depression and anxiety symptoms during pregnancy are significantly associated with children’s white matter microstructural development in the limbic system at age 8 years. These findings highlight the importance of prenatal mental health screening and potential interventions to promote brain development and support optimal neurodevelopmental outcomes in children.

## Data Availability

The raw data supporting the conclusions of this article will be made available by the authors, without undue reservation.

## Footnotes

^1^ https://github.com/dipy/dipy

^2^ https://github.com/daducci/AMICO

## Glossary

BDI-II: Beck Depression Inventory-II
STAI: State–Trait Anxiety Inventory
MRI: magnetic resonance imaging
DTI: diffusion tensor imaging
DKI: diffusion kurtosis imaging
NODDI: neurite orientation dispersion and density imaging
FA: fractional anisotropy
MD: mean diffusivity
AD: axial diffusivity
RD: radial diffusivity
MK: mean kurtosis
AK: axial kurtosis
RK: radial kurtosis
NDI: neurite density index
ODI: orientation dispersion index
FWF: free water fraction
ROI: region of interest
BMI: body mass index
MP-PCA: Marchenko-Pastur principal component analysis
EPI: echo planar imaging
TR: repetition time
TE: echo time
AMICO: accelerated microstructure imaging via convex optimization
DIPY: diffusion imaging in python
CGG: cingulum–cingulate gyrus
CGH: cingulum– hippocampus
FXC: Fornix Cres
UF: uncinate fasciculus
